# Carbene-catalytic enantioselective synthesis of chiral macrocycles

**DOI:** 10.1039/d5sc07411h

**Published:** 2025-10-29

**Authors:** Huikun Yan, Yuanyuan Zhu, Gongming Yang, Shuangxi Gu

**Affiliations:** a State Key Laboratory of Green and Efficient Development of Phosphorus Resources, Key Laboratory for Green Chemical Process of Ministry of Education, Hubei Key Laboratory of Novel Reactor and Green Chemical Technology, School of Chemical Engineering & Pharmacy, Wuhan Institute of Technology Wuhan 430205 China ygm18@tsinghua.org.cn shuangxigu@163.com; b School of Chemistry and Environmental Engineering, Wuhan Institute of Technology Wuhan 430205 China

## Abstract

Chiral macrocyclic units are not only prevalent in natural products, bioactive molecules, and functional compounds, but also play significant roles in synthetic and host–guest chemistry. Although extensive efforts have been devoted to constructing chiral macrocycles, few methods have been disclosed to date. Consequently, the rapid enantioselective construction of optically active macrocycles remains a formidable challenge. *N*-Heterocyclic carbene (NHC) catalysis, a highly successful organocatalytic approach, has emerged as a powerful tool for rapidly constructing complex molecular architectures. However, only recently has this strategy been applied to achieve enantioselective synthesis of chiral macrocycles. This review highlights recent advances in NHC-catalyzed enantioselective synthesis of chiral macrocycles—including centrally chiral, planar chiral, and inherently chiral macrocycles, thereby providing a timely overview and foundation for future research.

## Abstract 2

In this review, we summarized recent advances in the enantioselective construction of chiral macrocycles—including center-, planar-, and inherently-chiral systems—enabled by NHC organocatalysis.

## Graphical abstract

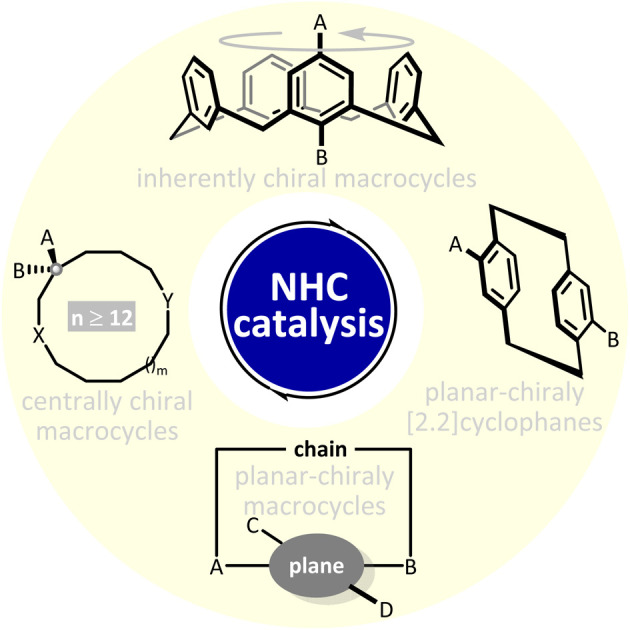

## 1 Introduction

**Fig. 1:**
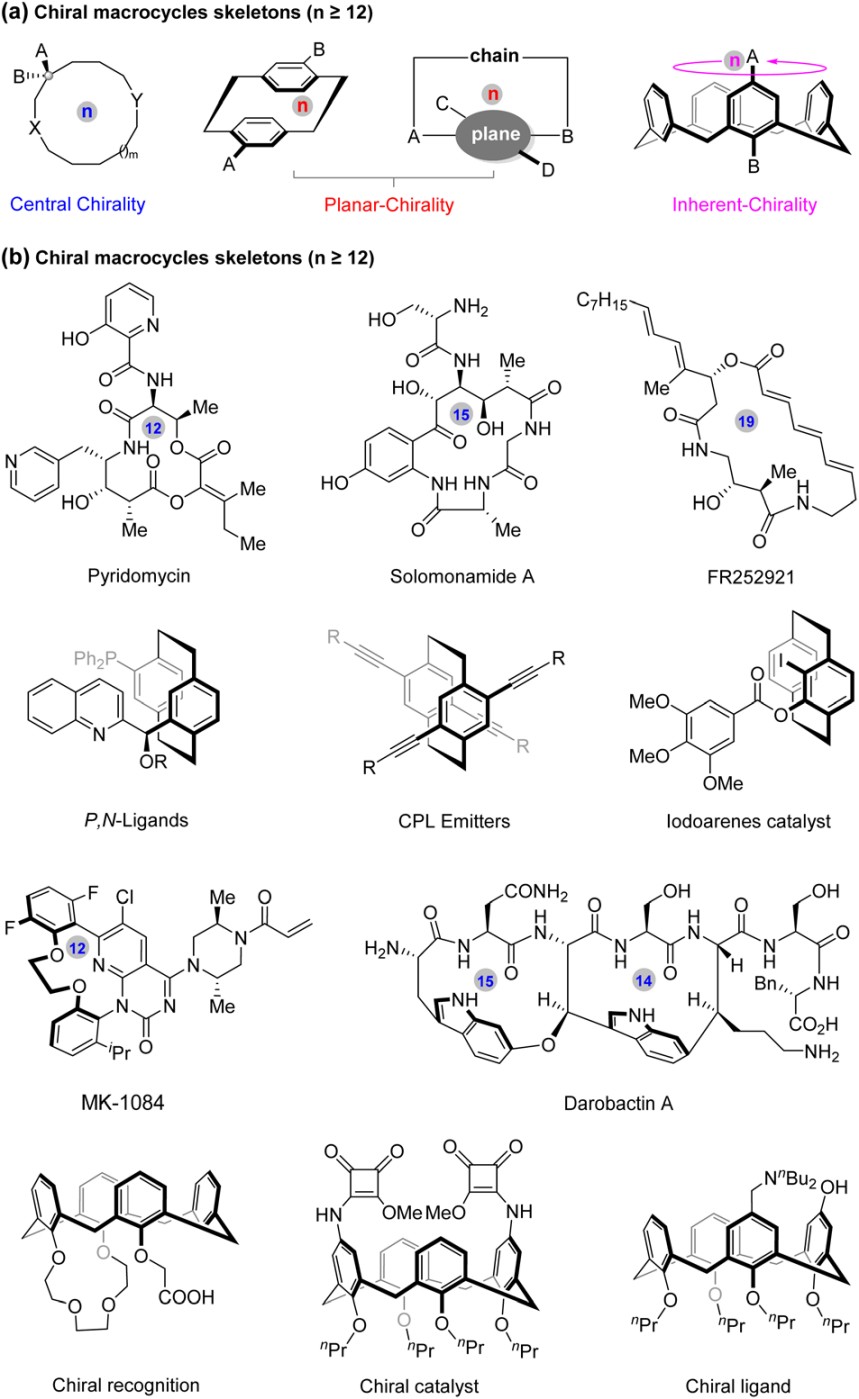
Representative molecules containing chiral macrocyclic moieties.

Chiral macrocyclic architectures (typically with more than eleven-membered rings) constitute privileged structural motifs prevalent in natural products,^1,2^ bioactive molecules,^3–5^ functional materials,^6^ as well as chiral organocatalysts and ligands.^7^ As shown in Fig. 1a, these chiral macrocycles can mainly be categorized into three types according to their asymmetric characteristics, including centrally, planar, and inherently chiral macrocycles. Notably, the planar chiral molecules include two types of planar chiral [2.2]paracyclophanes and planar chiral macrocycles.

In comparison with their corresponding linear analogues, the chiral macrocyclic molecules display distinct advantages due to their stable spatial conformations, which open avenues for scientists to explore new drugs, functional materials, as well as chiral catalysts and ligands.^8–12^ As outlined in Fig. 1b, centrally and planar chiral macrocyclic units have been widely found in many natural products and bioactive molecules,^13,14^ such as antituberculosis active pyridomycin, macrocyclic immunosuppressive agent FR252921,^15^ and planar chiral macrocyclic natural product darobactin A,^16,17^ which has selective inhibition against Gram-negative bacteria. Meanwhile, the planar chiral [2.2]paracyclophane skeletons have significant applications in materials science^18^ and asymmetric catalysis.^19^ Finally, the inherently chiral macrocyclic scaffolds, especially the calix[4]arenes, are of great importance in the area of enantioselective synthesis, chiral recognition,^20^ and host–guest chemisty.^21,22^ Owing to their widespread applications in drug discovery and synthetic chemistry, extensive efforts have been devoted to synthesizing enantiopure macrocycles. However, only limited methods have been reported to date for the assembly of chiral macrocycles when compared to the synthesis of other classes of chiral molecules. Overall, the rapid access to enantiomerically pure macrocyclic molecules in a highly enantioselective fashion is still in its infancy.

**Fig. 2:**
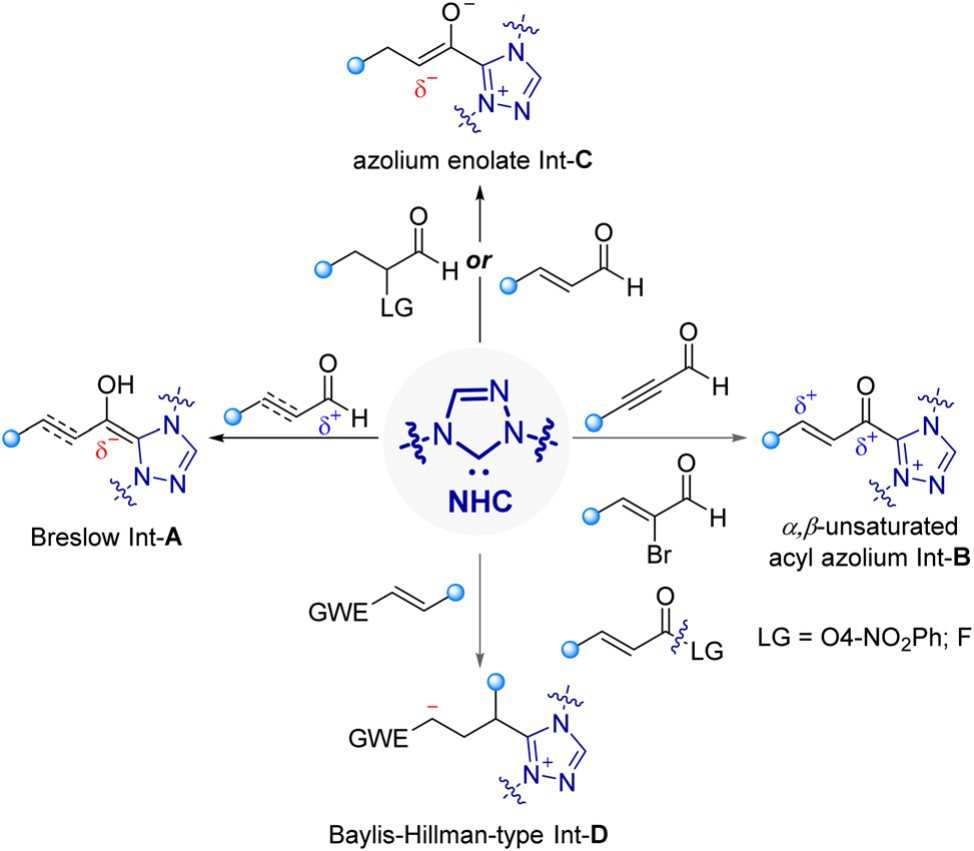
Representative NHC-bound reactive intermediates.

*N*-Heterocyclic carbene (NHC), one of the most successful organocatalysts, has been recognized to be a powerful tool for rapid construction of complex chiral scaffolds.^23–25^ Mechanistically, NHCs achieve highly enantioselective catalysis by taking advantage of their strong electron-donating ability, tunable steric environments, as well as capacity to generate diverse and reactive chiral intermediates (Fig. 2, such as Breslow, acyl azolium, azolium enolate, and Baylis–Hillman-type intermediates).^26–28^ Therefore, the NHC asymmetric catalysis has attracted extensive attentions from chemists and achieved significant developments in recent years. However, there are only very few methods have been reported to construct chiral macrocyclic systems to date when compared with the asymmetric preparation of other type of chiral molecules catalyzed by NHCs.

This review aims to summarize the recent progress in NHC-catalyzed enantioselective synthesis of chiral macrocycles, offering a critical overview of current research status while highlighting emerging strategies and providing future research directions in this rapid evolving field. In order to guide the reader through this topic, we categorize thesis reactions into four sections according to the asymmetric characteristics of the chiral macrocyclic products: (1) centrally chiral macrocycles; (2) planar chiral [2.2]-paracyclophanes; (3) planar chiral macrocycles; (4) inherently chiral macrocycles.

## 2 Enantioselective synthesis of chiral macrocycles *via* carbene catalysis

### 2.1 Centrally chiral macrocycles

Centrally chiral macrocyclic frameworks are not only highly important in scientific research and technology, but also prevalent in natural products and vital to medicinal applications.^29,30^ These macrocycles serve as versatile ligands and sensors with broad utility in supramolecular chemistry and self-assembly processes.^31,32^ However, macrocyclization remains challenging in current organic synthesis due to entropic penalties and transannular interactions during ring formation. Enantioselective macrocyclization is particularly difficult owing to the conformational flexibility of reaction intermediates. Therefore, the development of enantioselective access to optically pure macrocycles is still in its early stages.

**Fig. 3:**
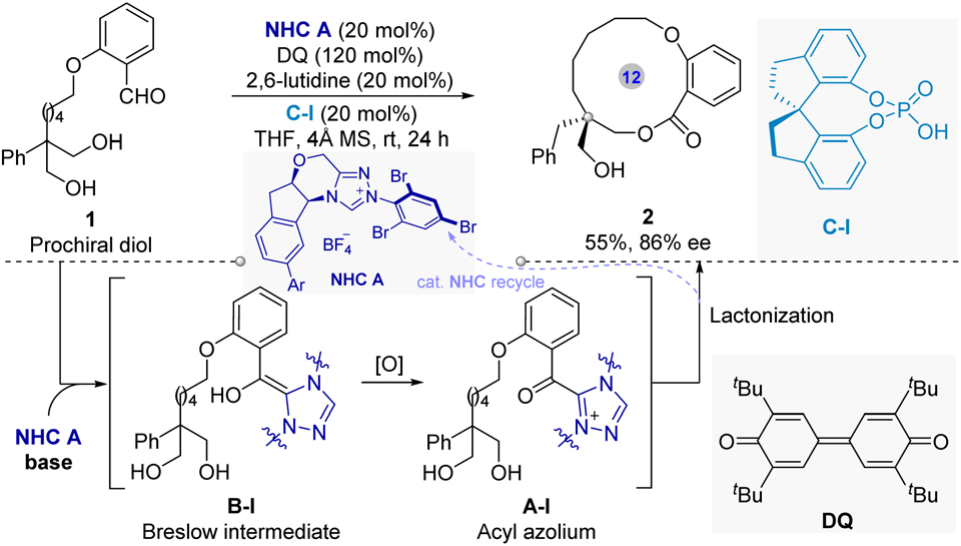
NHC-catalytic intramolecular enantioselective macrocyclization of 1,3-diol for the synthesis of centrally chiral macrolactone.

In 2016, Wang and colleagues^33^ pioneered a NHC asymmetric catalysis strategy for asymmetric synthesis of centrally chiral macrocycles. As shown in Fig. 3, the enantiopure macrolacton 2 was produced successfully in modern yield with high enantioselectivity *via* NHC-catalyzed intramolecular asymmetric macrocyclization of 1,3-diols 1. Furthermore, the optimization of the reaction conditions indicates that key to high enantioselectivity of this method was the employment of chiral phosphoric acid (CPA) C-I as a crucial cocatalyst, proposed to stabilize the transition state *via* hydrogen-bonding interactions.

Mechanistically, the catalytic cycle involves NHC addition to the aldehyde, forming a Breslow intermediate B-I, subsequent oxidation to generate a chiral acyl azolium specie A-I, then a highly enantioselective intramolecular esterification/macrocyclization by one of the 1,3-diol hydroxyl groups onto this active carbonyl, followed by the formation of the centrally chiral macrolactone and the release of catalyst to next catalytic cycle. Overall, this work represents the first highly catalytic enantioselective synthesis of centrally chiral macrocycles *via* NHC activation.

### 2.2 Planar-chiral [2.2]paracyclophanes

Planar chiral [2.2]paracyclophanes are composed of two benzene rings covalently linked by two ethylene bridges the *para*-position of the benzyl groups. These organic found tremendous use in asymmetric synthesis, as both ligands and catalysts,^34,35^ and in materials science, as polymers, energy materials and dyes.^7,18,36^ Nonetheless, enantioselective access to optically active [2.2]paracyclophanes still remains a long-standing formidable challenge in organic synthesis since its first discovery in 1949.^37^ Currently existing methods mainly rely on enantiomer separations or various resolutions with stoichiometric amounts of chiral reagents. Overall, rapid assembly of enantiopure [2.2]paracyclophane in a high enantioselective manner remains in its infancy.

#### 2.2.1 Kinetic resolution

**Fig. 4:**
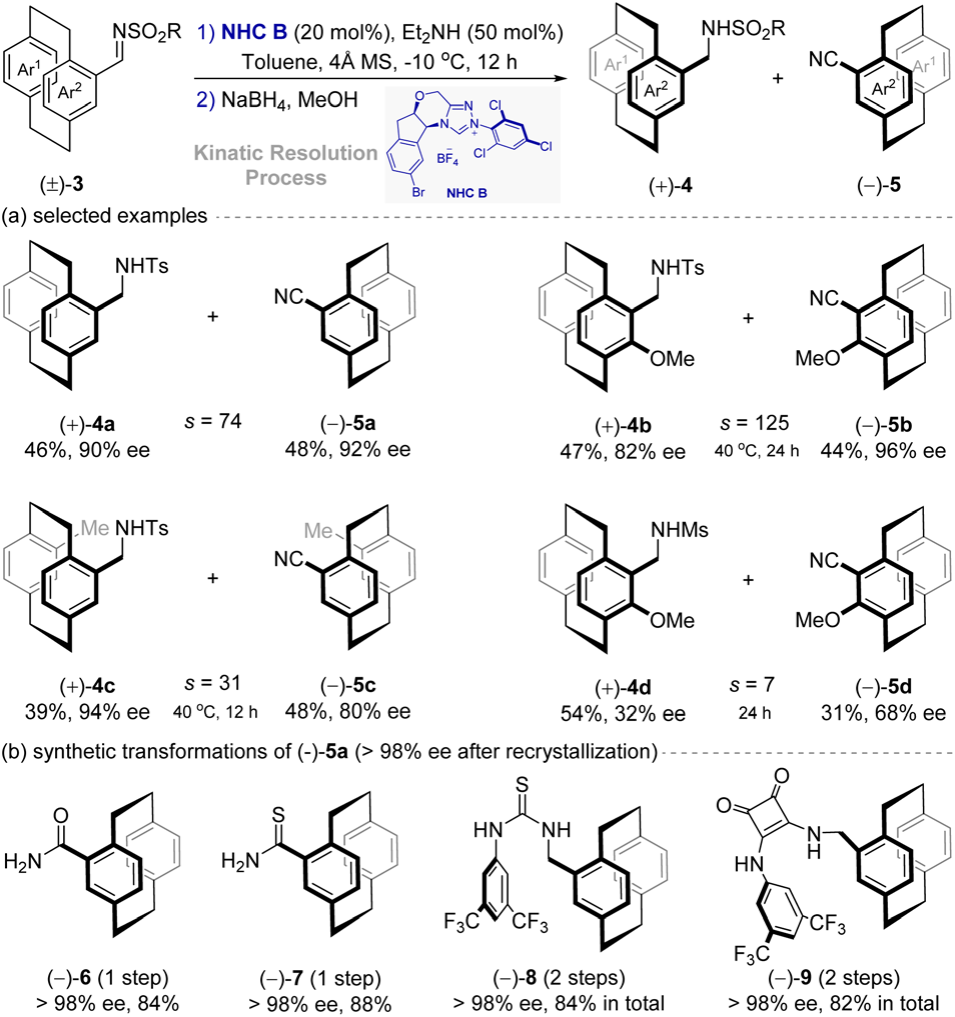
Asymmetric synthesis of planar chiral [2.2]paracyclophanes *via* NHC-catalytic kinetic resolution strategy.

In 2024, Chi, Jin and co-workers^38^ developed an NHC-catalyzed kinetic resolution strategy for synthesizing planar chiral [2.2]paracyclophanes (Fig. 4). Using a chiral aminoindanol-derived NHC B, racemic [2.2]paracyclophane inine substrates 3 underwent enantioselective desulfonylation or dehydrogenation. Key optimizations revealed that the solvent, substoichiometric base, and catalyst were critical for achieving high selectivity factors (*s* up to 264).

The reaction tolerated diverse substituents on the [2.2]paracyclophane rings and sulfonyl groups, affording both optically pure carbonitrile and sulphonamide products in excellent yields with enantioselectivities (up to >98% ee). Furthermore, the resulting planar chiral [2.2]paracyclophanes demonstrated significant synthetic utility and bioactivity. As shown in Fig. 4b, carbonitriles were derivatized to thioamides, amides, and primary amines without erosion of enantiopurity. Notably, five products exhibited superior antibacterial activity against *Xanthomonas oryzae* pv *Oryzae* (*Xoo*) compared to commercial pesticide thiodiazole copper (TC), highlighting their potential in agrochemical development.

#### 2.2.2 Parallel kinetic resolution

**Fig. 5:**
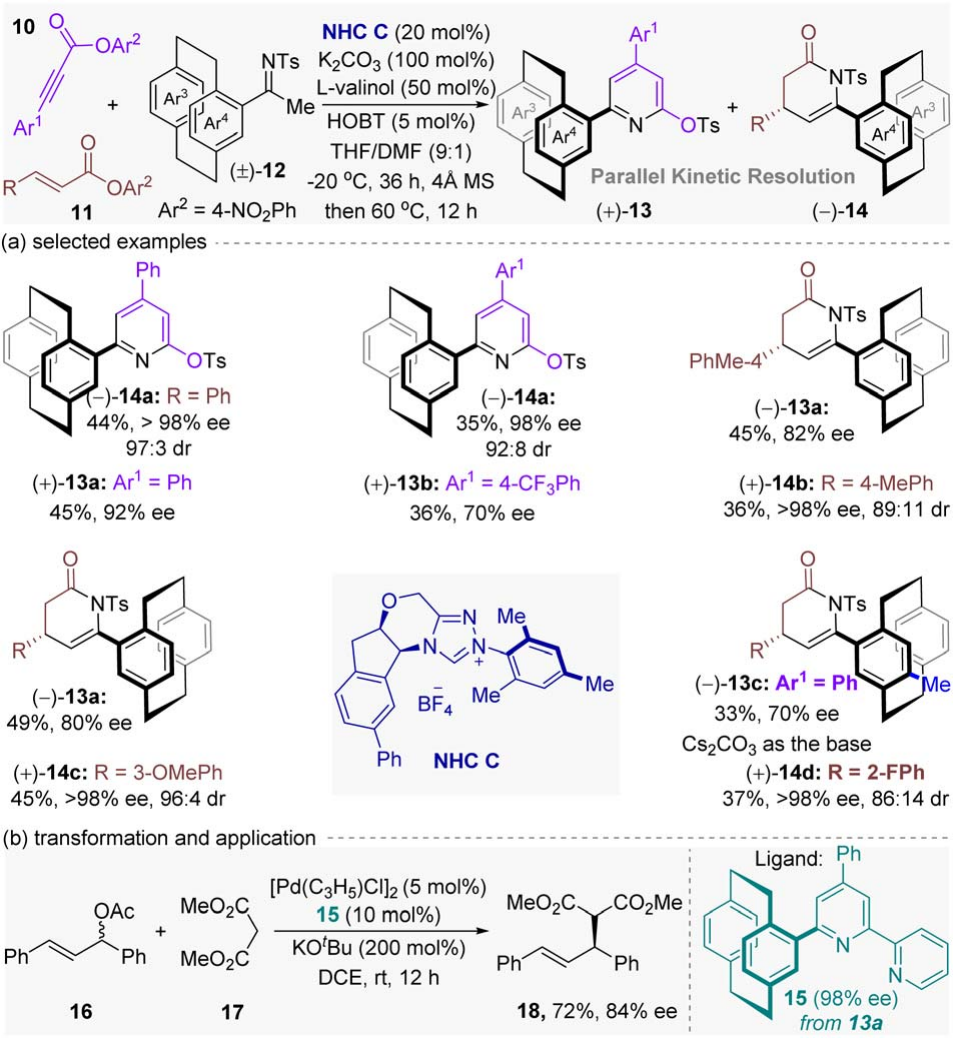
Asymmetric synthesis of planar chiral [2.2]paracyclophanes *via* NHC-catalytic chemodivergent parallel kinetic resolution strategy.

Soon later, the same group^39^ disclosed an unprecedented chemodivergent parallel kinetic resolution (PKR) of racemic planar chiral [2.2]paracyclophane ketimines using a single NHC catalyst (Fig. 5). Mechanistically, the NHC C activated two achiral esters (acetylenic esters 10 and cinnamic esters 11) generating distinct unsaturated acyl azolium intermediates. These intermediates enantioselectively reacted with opposite enantiomers of the racemic substrates (±)-12: (+)-12 formed enantioenriched trisubstituted pyridines (+)-13, while (−)-12 yielded γ-lactams (−)-14*via* [3 + 3] cycloaddition. Under the optimal conditions, this reaction proceeded smoothly with matched kinetics, affording both enantioenriched products (+)-13 and (−)-14 in excellent yields with high to excellent enantio-, and stereoselectivities (Fig. 5a, up to 98% ee, dr >97 : 3).

To evaluate the practicality of this protocol, a series of synthetic transformation experiments of the obtained planar chiral pyridines and lactams were performed. As illustrated in Fig. 5b, planar chiral product 13a (98% ee) underwent Ts-deprotection, chlorination, and triflation to access C-2 functionalized planar chiral [2.2]paracyclophane 15 without erosion of ee value. To further expand synthetic utility, the authors conducted a Pd-catalyzed asymmetric substitution reaction between the alkene 16 and the malonate 17 by using planar chiral [2.2]paracyclophane 15 as ligand, producing the desired chiral product 18 in 72% yield with 84% ee value. Additionally, the optically active (+)-14 displayed potent antibacterial activity against *Xoo* (71% inhibition at 100 μg mL^−1^), outperforming its *ent*-enantiomer and racemate, underscoring the significant role of planar chirality in bioactivity.

#### 2.2.3 Desymmetrization

**Fig. 6:**
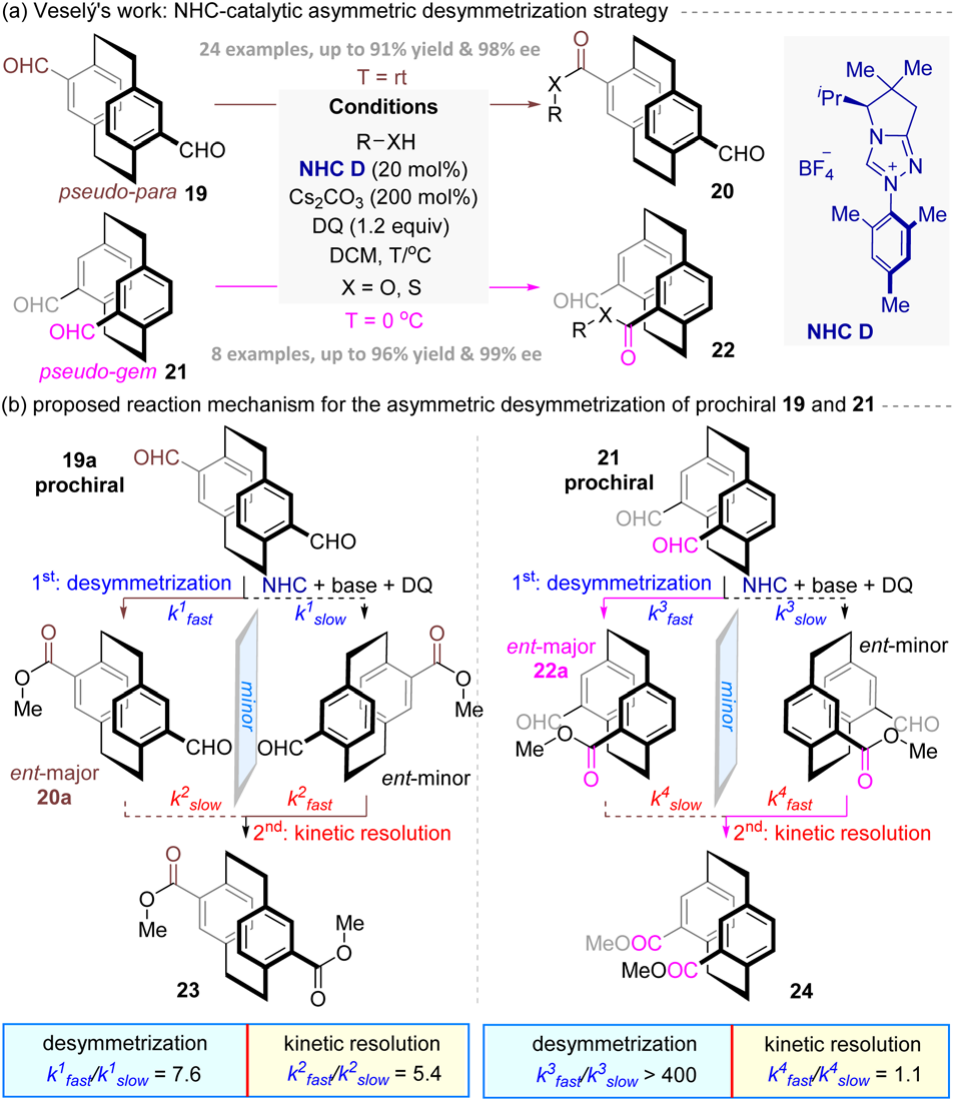
NHC-catalyzed asymmetric desymmetrization strategy for the synthesis of planar chiral [2.2]paracyclophanes.

Almost at the same time, Dočekal, Veselý and co-workers^40^ disclosed the first enantioselective desymmetrization of prochiral diformyl[2.2]paracyclophanes *via* NHC catalysis. As shown in Fig. 6, valine-derived triazolium precatalyst NHC D catalyzed enantioselective oxidative esterification of *pseudo-para* and *pseudo-gem* dialdehydes (19 and 21) with alcohols, yielding planar chiral monoesters (20 or 22). This methodology exhibited broad scope, accommodating aliphatic, aromatic, and bioactive alcohols, with thiols yielding thioesters. Furthermore, gram-scale synthesis of 22a (88% yield, 99% ee) underscored scalability. Additionally, the follow-up reactions of 22a, including Wittig olefination, reductive amination, and Pinnick oxidation, highlighted the usefulness of these planar chiral products.

Finally, to elucidate the reaction mechanism and origin of stereocontrol, a series of control experiments were performed. As summarized in the left of Fig. 6a, for prochiral substrate 19a, the reaction involved reversible Breslow intermediate formation (KIE = 2.8), followed by oxidation to form chiral acyl azolium intermediate, which then underwent a enantioselective esterification process to yield the major enantiomer 20a, with the reaction rate of the favoured enantiomer being 7.6 times faster than that of its *ent*-enantiomer. Subsequently, the *ent*-20a underwent the second esterification *via* a kinetic resolution pathway catalysed by the same catalyst with the 5.4 times reaction rate than the major product 20a. In contrast, prochiral 21 underwent irreversible Breslow formation (KIE = 0.5), enabling direct enantioselective desymmetrization (*k*_fast_^3^/*k*_slow_^3^ > 400) to afford the major enantiomer 22a in 91% yield with >99% ee value (Fig. 6b, right).

### 2.3 Planar-chiral macrocycles

Planar chiral macrocyclic frameworks are broadly used in drug discovery, asymmetric synthesis, and host–guest chemistry, and are also widely found in many natural products and bioactive molecules.^41^ Therefore, there are have attracted extensive attention from chemists and significant efforts have been devoted to synthesize enantiopure planar chiral macrocyclic molecules.^42–44^ However, due to their low racemization barrier and difficulty of predicting the occurrence of planar chirality, the catalytic enantioselective construction of planar chiral macrocycles is still in its infancy and remains a great challenge.

#### 2.3.1 Intramolecular macrocyclization

**Fig. 7:**
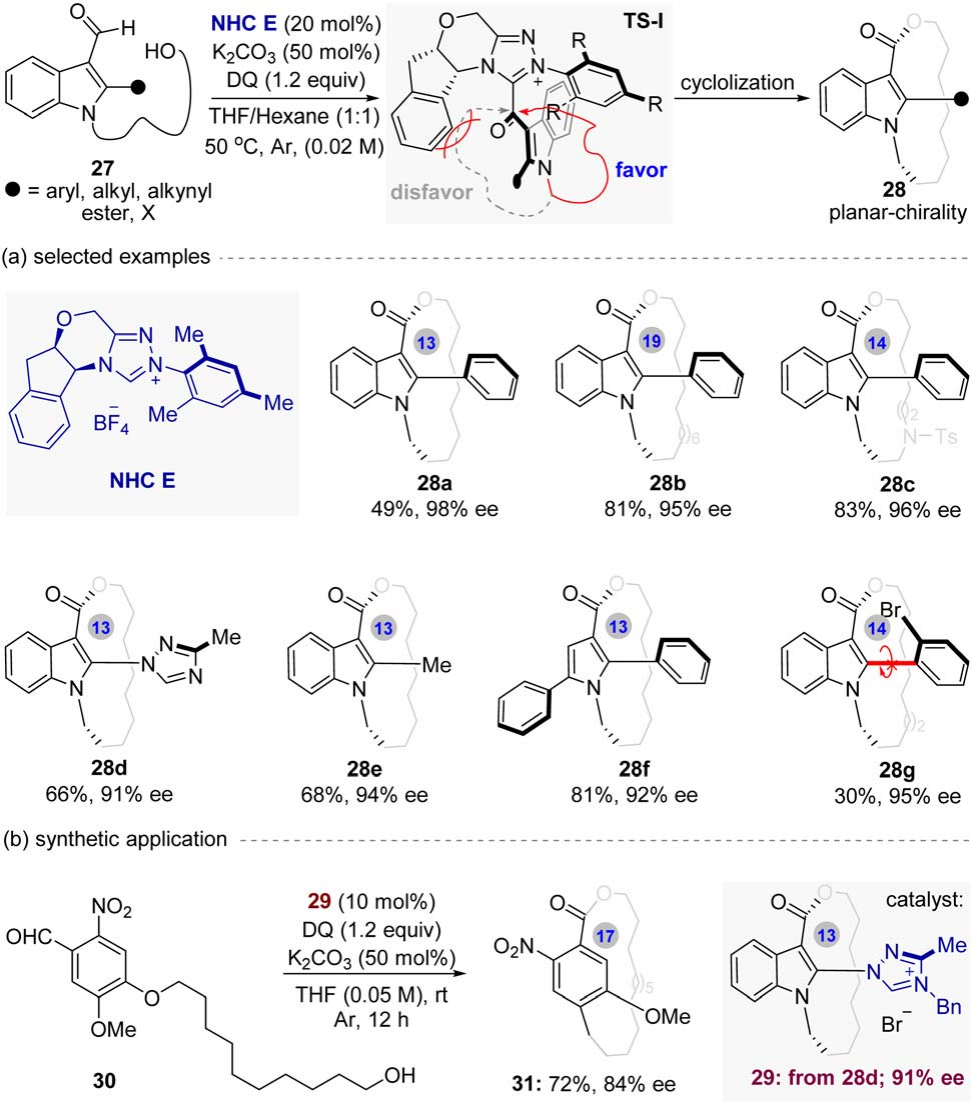
NHC-catalyzed intramolecular enantioselective macrocyclization for the synthesis of indole/pyrrole-based planar chiral macrocycles.

In 2024, with their ongoing interest in the organocatalytic synthesis of optically active atropisomers,^45–49^ the Wang and co-workers^50^ disclosed the first enantioselective construction of indole/pyrrole-based planar chiral macrocycles *via* NHC-catalyzed intramolecular macrocyclization of 3-carboxaldehyde indole/pyrrole derivatives (Fig. 7). A wide range of enantiopure indole/pyrrole-based planar chiral macrocycles bearing varied ring sizes (13- to 19-membered) and functional group-containing *ansa* chain were readily obtained *via* this method, with promising yields and excellent enantioselectivities (up to 84% yield and 99% ee). It is noteworthy that the 19-membered product 28a is the largest configurationally stable planar chiral *meta*-cyclophane known to be reported.

To understand the relationship between ring size and the configurational stability of the planar chiral product, the 20-membered and 21-membered macrocycles were synthesized under the optimal conditions separately. As results, they were detected with 0% ee and without planar chirality separately, clearly indicating that the macrocyclic planar chirality is highly dependent on the ring size. In addition, this method also had been successfully used to the construction of novel planar chiral macrocyclic skeletons bearing multiple stereogenic elements. A series of enantiopure macrocycles with both planar and axial chiralities were obtained with promising enantioselectivities and excellent diastereoselectivities *via* asymmetric kinetic resolution process under the optimal conditions.

Finally, the practicality of this method was also been demonstrated by further investigation on thermal studies and synthetic transformations of the planar chiral products. Notably, a novel chiral NHC catalyst 29 (91%)—derived from 28d, was able to catalyze the atroposelective macrocyclization of 30, providing the desired planar chiral paracyclophane 29 in good yield with high enantioselectivity (Fig. 7, 72% yield, 84% ee). Overall, this method not only represents the first realization of catalytic enantioselective access to planar chiral indole/pyrrole-based macrocycles, but also opens up a new avenue for development of NHC asymmetric catalysis.

**Fig. 8:**
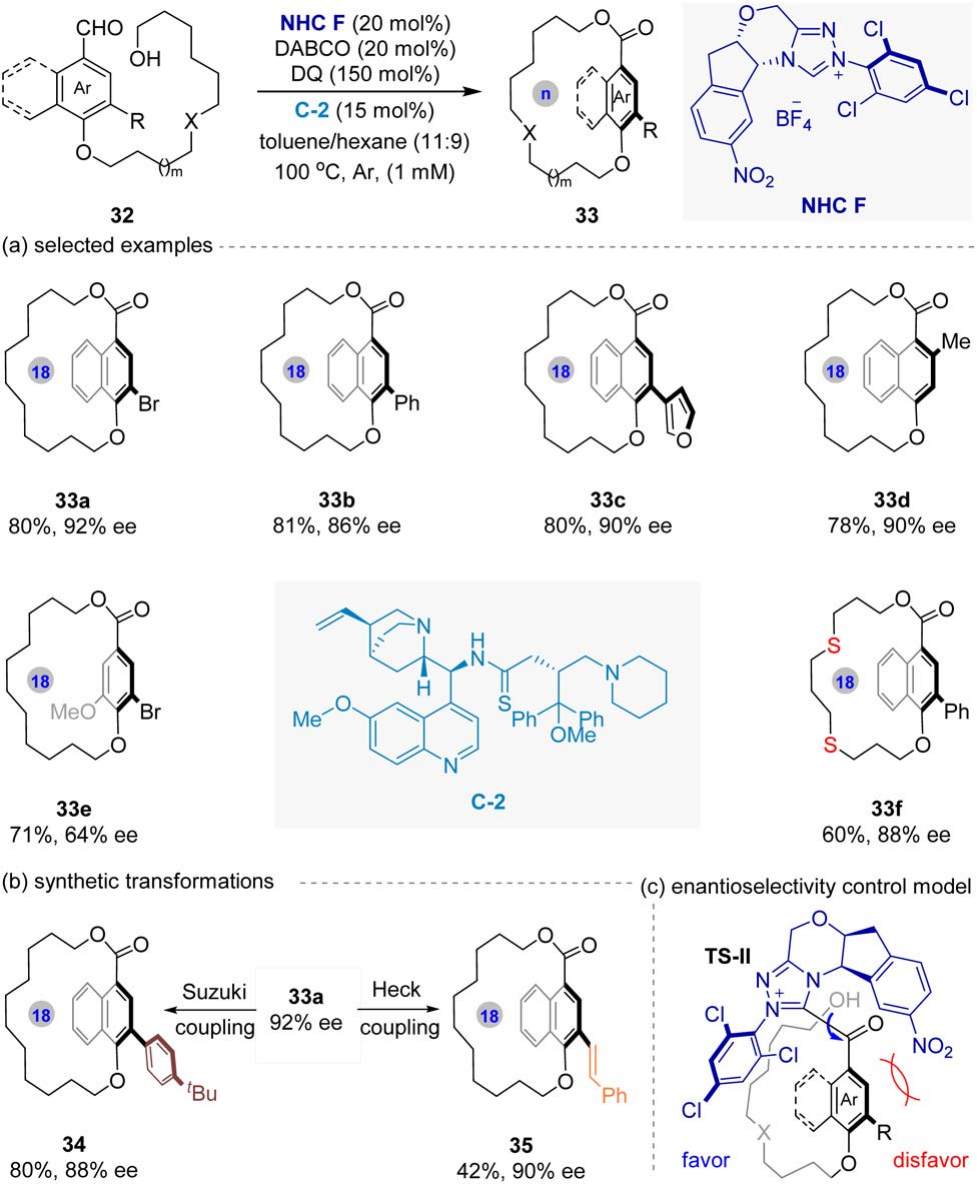
NHC-catalyzed intramolecular macrocyclization for the synthesis of planar chiral paracyclophanes (Chi's work).

Meanwhile, Chi and co-workers^51^ reported another NHC-catalyzed enantioselective macrocyclization strategy for the synthesis of planar chiral paracyclophanes from achiral bifunctional hydroxyl-aldehyde substrates 32 (Fig. 8). The plausible key intermediate TS-II is generated through the reaction of NHC with substrates 32 under oxidative conditions. This key specie simultaneously facilitates the macrocyclization *via* intramolecular esterification and dictates the planar chirality by differentiating the prochiral faces of aromatic ring within the substrate. Notably, the co-catalyst C-II plays a crucial role in the high yield and stereocontrol of this reaction. This method demonstrates broad scope, efficiently producing enantioenriched planar chiral paracyclophanes bearing varied functional group-containing both *anas* chain and aromatic plane in good-to-excellent yields (up to 82%) with high enantioselectivities (up to 92% ee).

In addition, the resulting planar chiral macrocycles exhibit significant configurational stability, resisting racemization even at elevated temperatures (150 °C). Finally, the utility of the products is highlighted by successful derivatization *via* transition-metal-catalyzed cross-coupling reactions (including Suzuki, Hech, Sonogshira), showcasing their potential as versatile chiral building blocks for further elaboration.

**Fig. 9:**
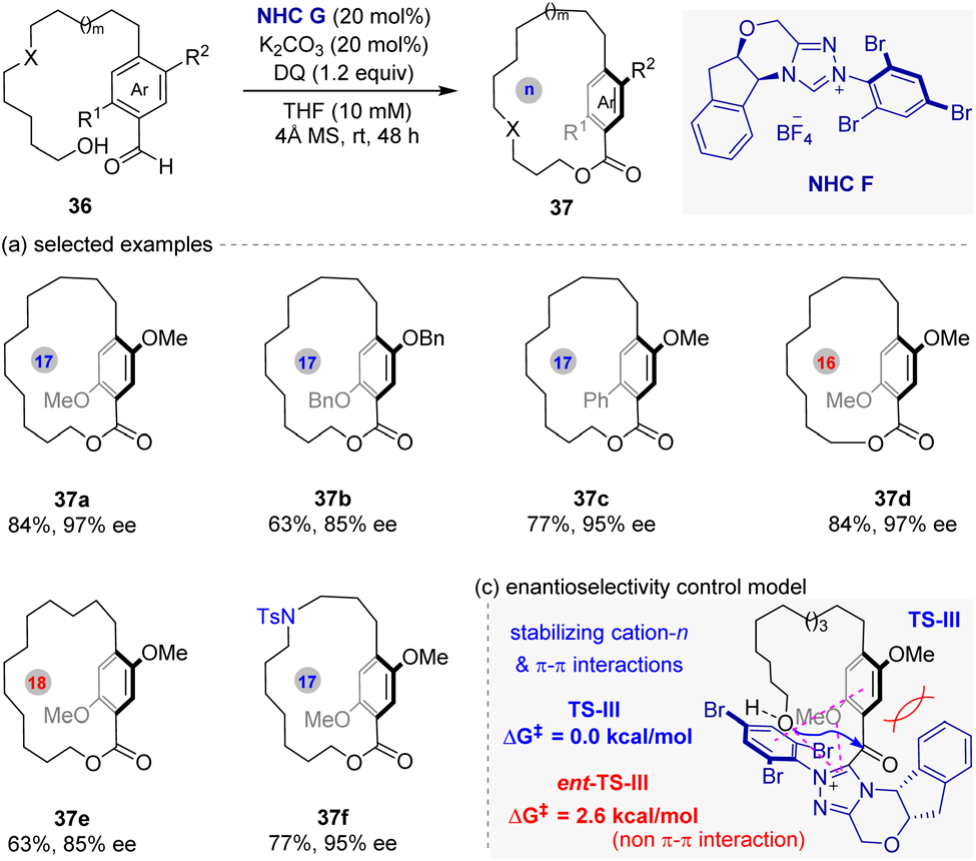
NHC-catalyzed intramolecular macrocyclization for the synthesis of planar chiral paracyclophanes (Zhao's work).

At the same time, Zhao and co-workers^52^ uncovered a similar strategy for the synthesis of planar chiral paracyclophanes (Fig. 9). The reaction utilizes bifunctional hydroxy-aldehyde substrates 36, where the NHC forms an acyl azolium intermediate TS-III under oxidative conditions, enabling intramolecular esterification to form the macrocycle while controlling planar stereogenicity. Crucially, high enantioselectivities (up to 99% ee) are achieved across diverse ring sizes (15- to 18-membered) and substituents on the aromatic moiety (aryl, heteroaryl, alkynyl). Furthermore, this method is also successfully applied to synthesize planar-chiral derivatives of pharmaceuticals (including gemfibrozil, telmisartan, indomethacin, *etc.*) and peptides. Despite above broad substrate scopes, the type of these substrates remains limited to the *ortho*-alkoxy group substituted aromatic aldehydes.

Finally, the origin enentiocontrol in this process was also elucidated by controlled experiments and density functional theory (DFT) calculations. As shown in Fig. 9c, the results indicate that the enentioselectivity primarily stems from a stabilizing cation–π interaction between the electron-deficient acyl azolium and electron-rich OR groups (*e.g.*, methoxy groups) within the substrate. An additional π–π interaction between the substrate's aromatic ring and the NHC's *N*-aryl substituent further stabilizes the favored transition state.

**Fig. 10:**
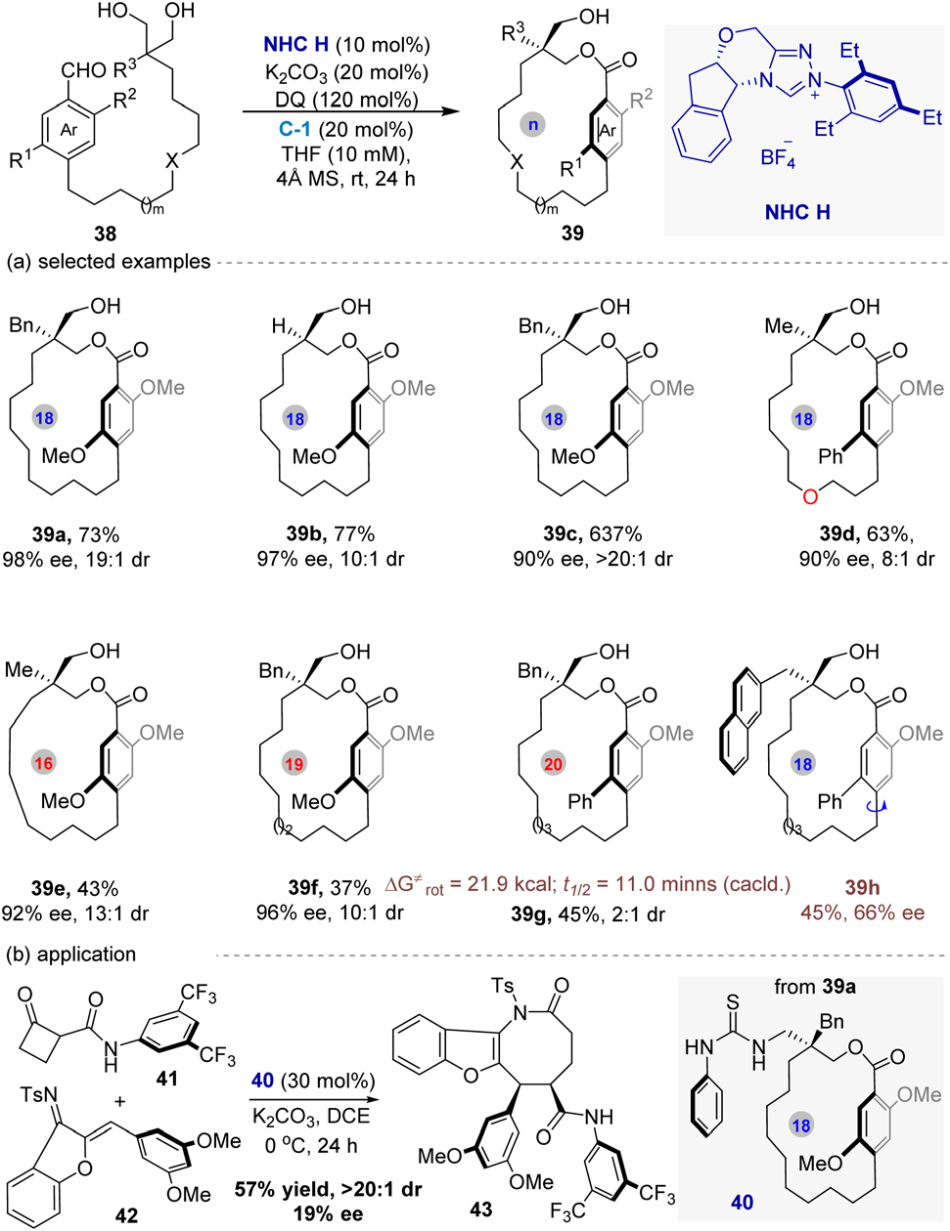
Enantio-, atrop-, and diastereoselective intramolecular macrocyclization for the synthesis of type III planar chiral cyclophanes enabled by NHC and CPA co-catalysis.

Recently, following their continued interest in asymmetric construction of planar chirality,^53–57^ the Zhao group^58^ disclosed the first enantio-, atrop-, and diastereoselective macrocyclization enabled by the NHC and CPA co-catalysts, yielding type III planar chiral cyclophanes featuring chiral *ansa* chains (Fig. 10). This strategy centers on the NHC-catalyzed desymmetrization of prochiral 1,3-diols embedded within linear precursors. The NHC generates an acyl azolium specie that initiates macrocyclization, while the CPA co-catalyst engages the diols and acyl azolium intermediates *via* hydrogen bonding, critically enhancing the diastereoselectivity of the process.

Under the optimal conditions, a range of optically active macrocycles with varying ring sizes (17- to 19-membered) and diverse aromatic ring substituents (aryl, heteroaryl) were achieved effectively *via* this method. In addition, the thermodynamic studies and DFT calculations demonstrate that the chiral substituent significantly increases the rotational barrier of the benzene ring within the macrocycle compared to unsubstituted analogues. Computational analysis reveals that the chiral substituent shrinks the *ansa* chain by compressing the bond angle, thereby hindering the conformational rotation responsible for racemization. Finally, the use of 40—derived from 39a, in the asymmetric [4 + 4]cycloadditon as the organocatlyst demonstrates the potential utility of these type III planar chiral macrocycles (Fig. 10b). Overall, this work provides a catalytic alternative to substrate-controlled diastereoselective macrocyclizations for accessing Type III cyclophanes.

#### 2.3.2 Intermolecular macrocyclization

**Fig. 11:**
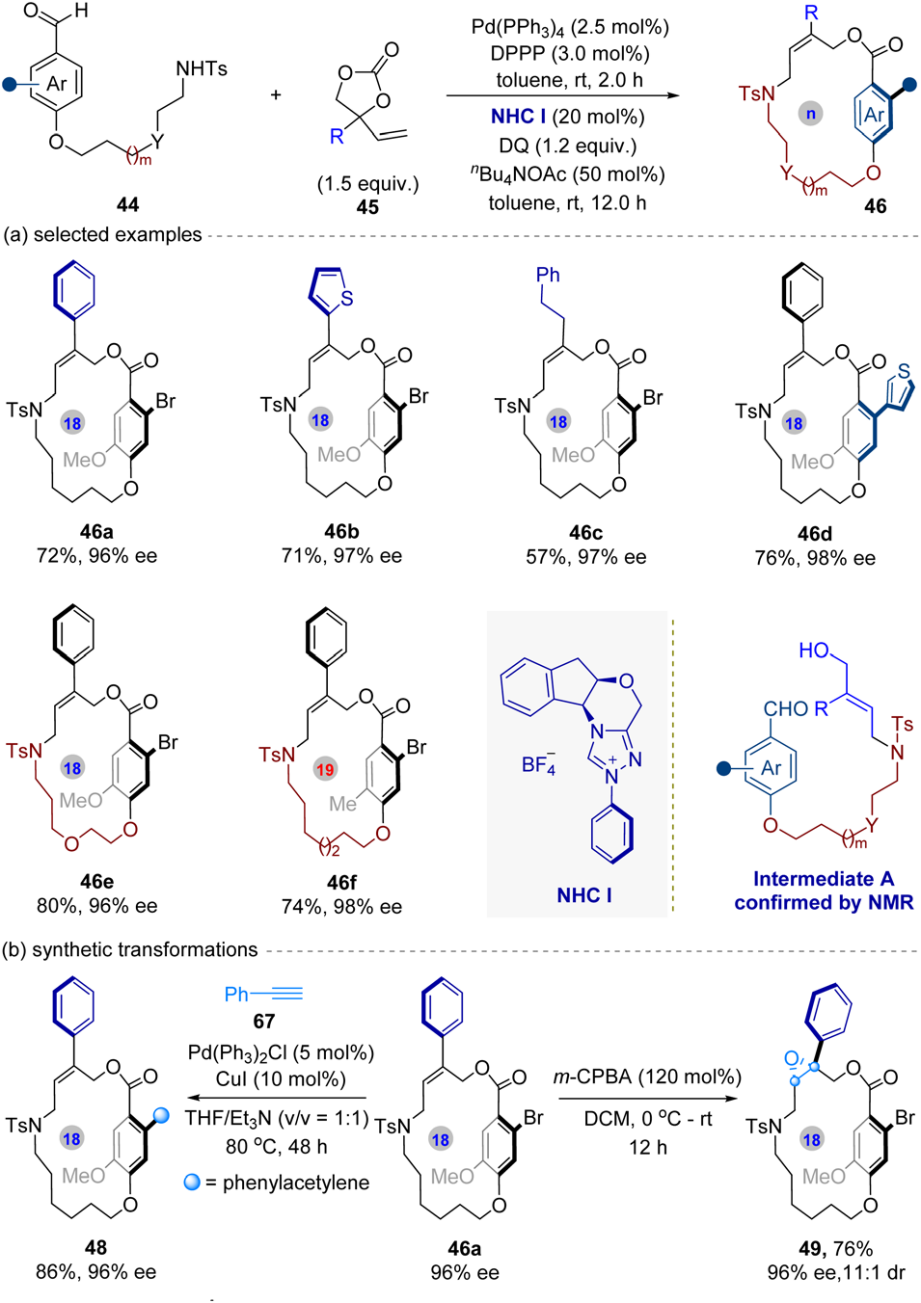
Pd/NHC sequentially catalyzed intermolecular atroposelective macrocyclization for the synthesis of planar chiral macrocycles.

In 2024, the Wang group^59^ introduced a pioneering sequential Pd/NHC catalytic system for the intermolecular atroposelective synthesis of planar chiral macrocycles (Fig. 11). To address the limited synthetic accessibility of such structures, the authors combined Pd catalysis with NHC organocatalysis in one-pot process. In this sequentially catalytic process, the Pd(0) catalyst first mediates the reaction between hydroxybenzaldehyde derivatives (44) and vinyl ethylene carbonates 45 (VECs), forming the allylic alcohol intermediates A—confirmed by NMR analysis. Subsequently, the NHC catalyst attacks on the aldehyde group of intermediates A to form Breslow intermediates, which undergo oxidation to afford acyl azoliums. Finally, the acyl azoliums undergo esterification to give desired planar chiral products 46, releasing the NHC catalyst for next catalytic cycle.

This method features broad substrate scope and high functional group tolerance. For instance, the aryl- and heteroaryl-substituted VECs delivered corresponding macrocycles (46a–46b) with excellent enantioselectivities, while alkyl-substituted VECs required longer reaction times but maintained promising stereocontrol (46c). The aryl aldehyde scope accommodated electron-donating/withdrawing groups and heterocycles. Crucially, macrocycle ring size dictates planar chirality: 17- to 19-membered rings retained chirality, while 20-membered analogues racemized due to low rotational barriers. Furthermore, racemization studies confirmed high thermal stability of these obtained planar chiral macrocycles. In addition, gram-scale synthesis and derivatizations (Fig. 11b, *e.g.*, Sonogashira coupling, epoxidation) underscored synthetic utility. Stepwise experiments validated the sequential mechanism, distinguishing this strategy from intramolecular approaches reliant on *pre*-functionalized substrates.

#### 2.3.3 Dynamic kinetic resolution

**Fig. 12:**
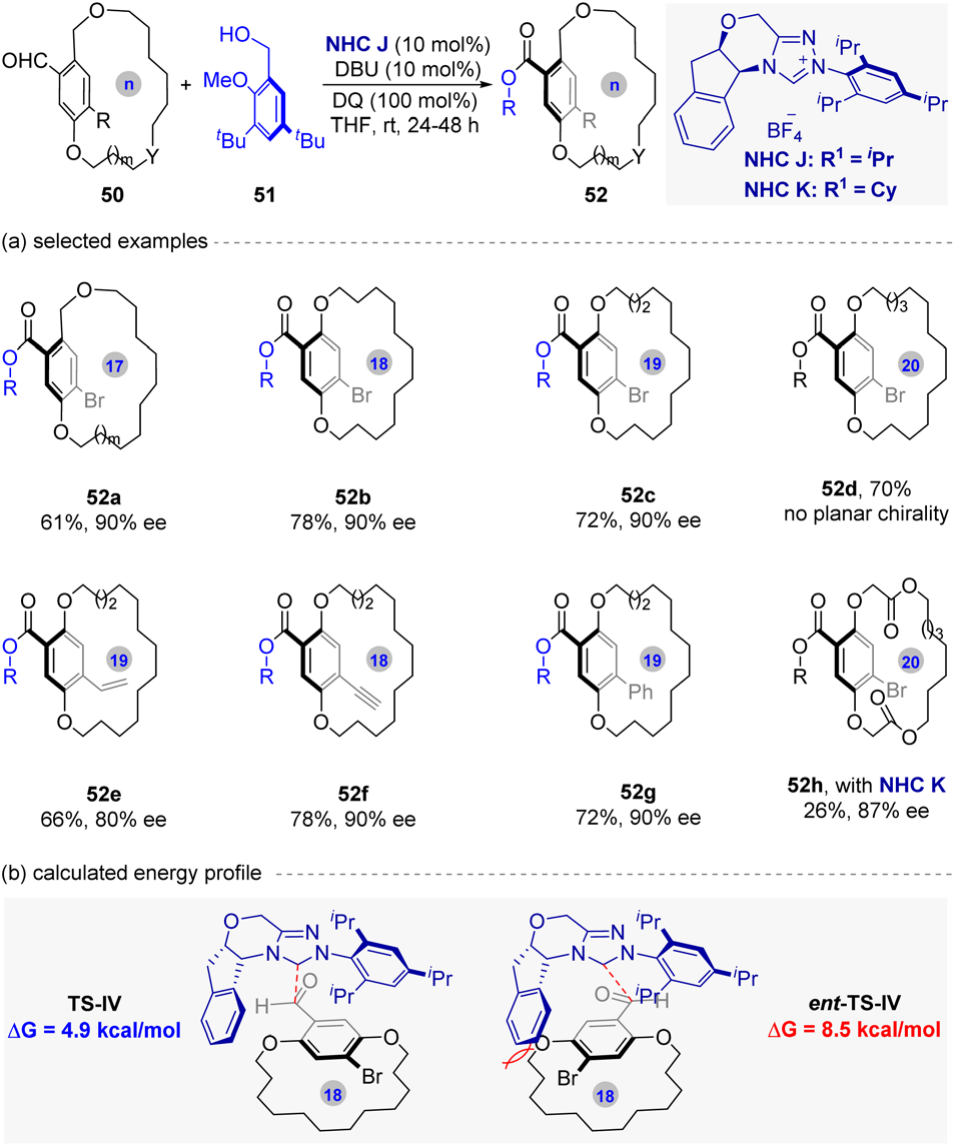
NHC-catalyzed asymmetric DKR strategy for the synthesis of planar chiral macrocycles.

In 2024, Zhao and co-workers^60^ disclosed a NHC-catalyzed dynamic kinetic resolution (DKR) strategy for the synthesis of planar chiral macrocycles (Fig. 12). Using racemic macrocyclic dialdhydes 50 and alcohols 51, the reaction achieved enantioselective esterification *via* NHC catalysis under oxidative conditions, affording the desired planar chiral products 52 in up to 97% yield and >99% ee value.

Based on the experimental results of the substrate scope, a central insight is the critical balance between ring size and aryl substituent size for effective DKR. While 17- to 19-membered substrates underwent efficient DKR, smaller sizes (*e.g.*, 11- to 12-membered) led to kinetic resolution due to slow racemization, and bigger size (20-membered) with flexible *ansa* chain (52d) abolished planar chirality. In addition, the utility of this method was also been demonstrated by the diversified derivatizations of the products. Mechanistic studies confirmed rapid substrate racemization (KIE ≈ 1) and identified NHC addition as the enantiodetermining step. As shown in Fig. 12b, the DFT calculations revealed a 3.6 kcal mol^−1^ energy difference between transition states TS-IV and its enantiomer (*ent*-TS-IV), rationalizing selectivity. Furthermore, these rigid products exhibited exceptional configurational stability (no racemization after 7 days at 110 °C in toluene), highlighting their potential as chiral scaffolds.

### 2.4 Inherently chiral macrocycles

Inherently chiral system, featuring asymmetric spatial arrangements around curved surfaces lacking vertical symmetry planes, occur extensively in bioactive compounds and functional materials.^61–64^ Among these, inherently chiral macrocycles, particularly calix[4]arenes, are prominently employed in host–guest chemistry, asymmetric catalysis, and chiral recognition applications.^65–68^ However, despite these potential applications, the enantioselective construction of such scaffolds is still a big challenge and only few methods have been disclosed because of their special spatial distortion configuration. In addition, these reports mainly focus on the transition-metal-catalyzed asymmetric transformations or kinetic resolution of prochiral/racemic macrocycles. Overall, rapid synthesis of inherently chiral macrocycles in highly enantioselective fashion is still in its infancy.

#### 2.4.1 Desymmetrization

**Fig. 13:**
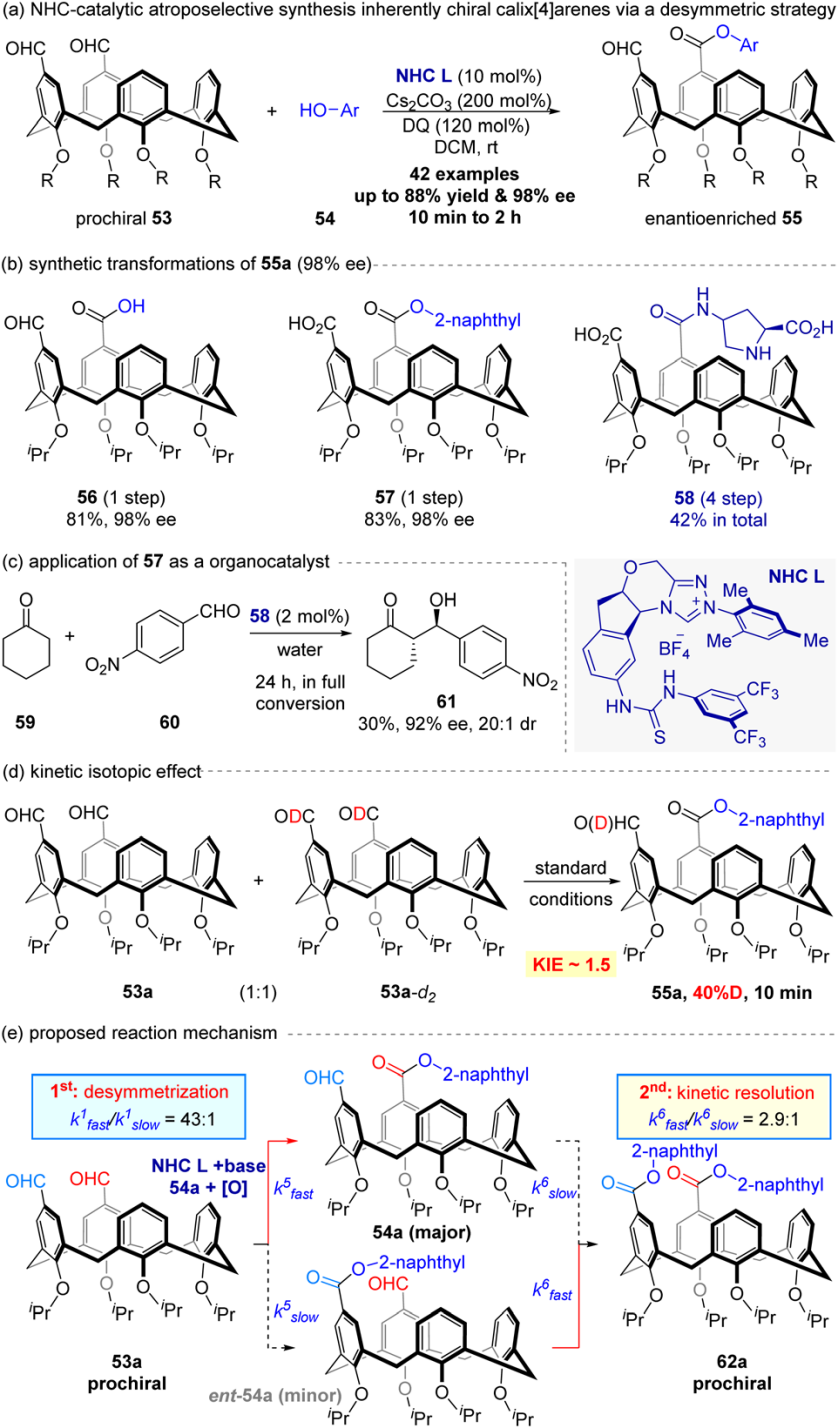
NHC-catalyzed asymmetric desymmetrization strategy for the synthesis of enantioenriched calix[4]arens with inherent chirality.

Recently, Dočekal, Veselý and co-workers^69^ developed a bifunctional NHC-catalyzed desymmetrization of prochiral diformylcalix[4]arenes (53) to access inherently chiral macrocycles (Fig. 13). The thiourea-functionalized NHC L enabled enantioselective esterification with phenols (54), yielding corresponding inherently chiral calix[4]arenes 55 in up to 88% yields and 98% ee within 2.0 hours. The bifunctional design leverages hydrogen bonding to stabilize the Breslow intermediate, crucial for enantiocontrol.

This reaction exhibits exceptional scope and practicality. Lower-rim alkyl groups and diverse phenols—including electron-rich/deficient arenes, natural products (*e.g.*, eugenol, capsaicin), and pharmaceuticals (*e.g.*, estrone, ezetimibe)—delivered products with promising ee values. Gram-scale synthesis (55a, 79% yield, 98% ee) and post-functionalizations (Wittig olefination, reduction, Pinnick oxidation) afforded versatile chiral building blocks without erosion of optical purity (Fig. 13b, 56–58). Furthermore, the carboxylic acid derivative 58 served as a precursor for hybrid organocatalysts (Fig. 13c), which facilitated an aqueous aldol reaction (30% yield, 92% ee).

Mechanistic studies using deuterium labeling and KIE measurements (Fig. 13d, KIE = 1.5) indicate a rate-determining 1,2-proton shift in Breslow intermediate formation. Control experiments confirme desymmetrization governs enantioselectivity (for model reaction: *k*_fast_^1^/*k*_slow_^1^ = 43 : 1), not kinetic resolution, and a postulated mechanistic pathway is presented in Fig. 13e. Overall, this metal-free protocol offers a valuable platform for asymmetric molecular recognition and catalysis.

#### 2.4.2 (Dynamic) kinetic resolution

**Fig. 14:**
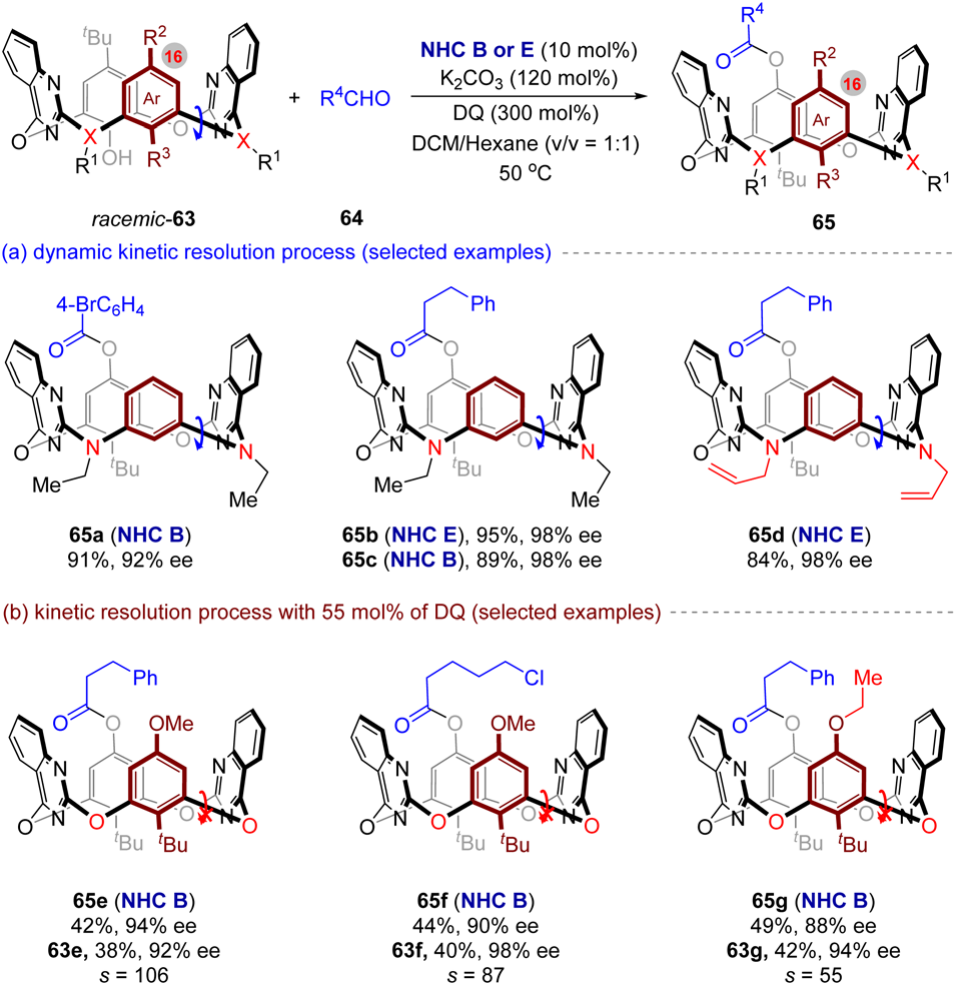
NHC-catalyzed enantioselective (dynamic) kinetic resolution for synthesis of macrocycles with inherent chirality.

Very recently, Wang and co-workers^70^ uncovered the first NHC-catalyzed enantioselective synthesis of inherently chiral macrocycles *via* (dynamic) kinetic resolution strategies (Fig. 14). Under optimal conditions, this reaction produces the corresponding enantiopure heteroatom-bridged calix[4]arenes 65 smoothly with promising yields and enantioselectivities.

Meanwhile, the key optimizations reveal elevated temperature accelerated substrate racemization (critical for DKR process), while diluted conditions enhanced enantiocontrol. The DKR reaction demonstrates broad scope across aromatic/heteroaromatic aldehydes and alkyl aldehydes, with the latter showing superior compatibility (Fig. 14a, 65a–65d). On the other hands, for configurationally stable macrocycles (Fig. 14b, 63e–63g), KR process affords enantiomerically enriched products (65e–65g) and recovered starting materials with high selectivity factors (*s* up to 106).

Furthermore, the synthetic utility of this protocol has been demonstrated by the gram-scale synthesis of 65c and a range of its post-functionalization (such as sonogashira coupling). Finally, the origins of the high enantioselectivity for the DKR process have been elucidated by DFT calculations. Overall, this work pioneers NHC-catalytic (D)KR strategies for the synthesis of inherently chiral macrocycles, offering robust access to enantiopure calix[4]heteroarenes, and providing a valuable platform for supramolecular chemisty.

## 3 Conclusions and outlooks

Chiral macrocyclic frameworks are not only prevalent in many natural products, bioactive molecules, and functional compounds, but also play critical roles in asymmetric catalysis and host–guest chemistry. Although they have attracted significant attention and extensive efforts have been devoted to synthesizing enantiopure macrocycles, only limited methods have been reported, and development is still in its early stage. Meanwhile, NHC catalysis has emerged as a versatile platform for the asymmetric construction of complex molecular architectures. However, only recently has this strategy been employed to constructing chiral macrocycles. These remarkable achievements are significant both for advancing NHC asymmetric catalysis and for enabling unprecedented methods to construct structurally diverse chiral macrocycles.

Despite these progresses, NHC-catalytic enantioselective synthesis of chiral macrocycles is still in its infancy, and there remains several challenges and opportunities merit exploration: (1) design and construction of novel chiral macrocycles with multiple stereogenic elements—such as with both axial and planar chiralities, or with heteroatom stereogenic centers—including nitrogen-, and phosphorus-stereocenters.^71^ A broad range of natural products and bioactive compounds feature multiple stereogenic elements, which have significant influences on their biological and physical properties.^72^ (2) Exploration of new catalysts and novel catalytic mechanisms, which is highly desirable for advancing the field and is particularly important for improving stereocontrol. (3) Development of new strategies for NHC catalytic catalysis—such as integration with photochemical, electrochemical, or biocatalytic approaches. Exploring greener catalytic schemes would address the scalability and sustainability issues, and will be crucial for future industrial transformations. Overall, this review aims to offer a panoramic overview of research progress, challenges, and opportunities in catalytic enantioselective synthesis of chiral macrocycles enabled by NHC catalysis for potential researchers. We tentatively conclude that further exploration will foster sustainable developments in this burgeoning field.

## Author contributions

H. Y. and Y. Z. prepared the manuscript with contributions from all the co-authors. All authors revised the manuscript.

## Conflicts of interest

The authors declare no conflict of interest.

## Data Availability

No primary research results, software or code have been included and no new date were generated or analysed as part of this review.
